# Characteristics and Antibiotic Preferences of US Adults Reporting Frequent Use vs No Use of Antibiotics

**DOI:** 10.1001/jamanetworkopen.2025.1429

**Published:** 2025-03-21

**Authors:** Alistair Thorpe, Rachael A. Lee, Angela Fagerlin, Valerie M. Vaughn, Julia E. Szymczak

**Affiliations:** 1 Department of Population Health Sciences, Spencer Fox Eccles School of Medicine at University of Utah, Salt Lake City; 2 Department of Medicine, Division of Infectious Diseases, UAB School of Medicine, Birmingham, Alabama; 3 Salt Lake City VA Informatics Decision-Enhancement and Analytic Sciences (IDEAS) Center for Innovation, Salt Lake City, Utah; 4 Department of Internal Medicine, Division of General Internal Medicine, Spencer Fox Eccles School of Medicine at University of Utah, Salt Lake City; 5 Department of Internal Medicine, Division of Epidemiology, Spencer Fox Eccles School of Medicine at University of Utah, Salt Lake City

## Abstract

This survey study compares demographic characteristics, beliefs, and antibiotic preferences of US adults who frequently use antibiotics with those who have not used antibiotics over the past year.

## Introduction

Antibiotic overuse can harm patients and has led to a public health crisis of antibiotic resistance.^1^ Despite such harms, some patients still seek antibiotics when unnecessary.^2^ Research suggests that certain patient characteristics and cognitive biases are associated with antibiotic-seeking behaviors and unnecessary exposure.^2,3^ Understanding differences between adults with high and low antibiotic use may enable clinicians to tailor messaging and education strategies to better engage patients and provide evidence-based care.^4^ We compared demographic characteristics, cognitive beliefs, and antibiotic preferences of US adults aged 18 to 64 years based on their self-reported exposure to antibiotics in the 12 months prior to the study (none vs 3 or more times).^5^

## Methods

This study analyzed results from an online survey of US adults about antibiotic use (completion rate, 89.8%). The survey and methods are available in Supplement 1. From March to April 2024, respondents were recruited by Dynata for this English-language survey. The University of Utah institutional review board deemed the study exempt and granted a waiver of informed consent due to minimal or no risk to respondents. The study followed STROBE reporting guidelines.

Respondents first saw a scenario describing symptoms of a viral respiratory infection where antibiotics are not indicated before reporting their antibiotic preferences (ie, likelihood of wanting antibiotics and concerns about adverse effects). Respondents then reported demographics and cognitive beliefs such as medical maximizing^6^ (ie, preferences for active vs passive approaches to health care).

Adults aged 18 years or more, residing in the US, and English-speaking were included. Quotas for self-reported age, gender identity, racial and ethnic identity, and US Census region were used to oversample underrepresented groups. For this analysis, adults 65 years or older were excluded as they were asked different age-specific questions for an unrelated study. In order to enhance data quality, we excluded respondents post hoc who finished within 5 minutes and those whose open-text answers raised doubts about the validity of their responses.

Survey respondents were classified, based on self-reported antibiotic use in the prior 12 months, into nonusers (ie, no antibiotic use) vs frequent use (ie, antibiotics taken 3 or more times).^5^ We used multiple logistic regression to explore differences in demographic characteristics and cognitive beliefs between groups. Using a hierarchical approach, we then included respondents’ likelihood of wanting antibiotics for a hypothetical viral respiratory infection and concerns about adverse effects to the original model. Tests were 2-sided with α = .05 and calculated using R version 4.2.2 (R Project for Statistical Computing).

## Results

Analyses include the 581 respondents aged 64 years or younger who completed the survey. The mean (SD) age was 42.6 (13.4) years; 332 respondents (57.1%) identified as women (Table).

**Table.** Respondent Characteristics and Antibiotic Preferences According to Self-Reported Antibiotic Use^a^

Characteristics	Antibiotic use in past 12 mo, respondents, No. (%)		Total respondents (n = 581)	*P* value^b^
	Nonusers (0 times) (n = 458)	High frequency (≥3 times) (n = 123)		
Age, y				
Mean (SD)	44.2 (13.1)	36.9 (12.7)	42.6 (13.4)	<.001
18 to 33	120 (26.2)	61 (49.6)	181 (31.2)	<.001
34 to 49	150 (32.8)	39 (31.7)	189 (32.5)	
50 to 64	188 (41.0)	23 (18.7)	211 (36.3)	
Gender				
Men	187 (40.8)	55 (44.7)	242 (41.7)	.42
Women	267 (58.3)	65 (52.8)	332 (57.1)	
Transgender woman	0	1 (0.8)	1 (0.2)	
Transgender man	1 (0.2)	0	1 (0.2)	
Nonbinary or third gender	1 (0.2)	1 (0.8)	2 (0.3)	
Prefer not to say	2 (0.4)	1 (0.8)	3 (0.5)	
Race and ethnicity				
Hispanic	157 (34.3)	39 (31.7)	196 (33.7)	.03
Non-Hispanic Black	159 (34.7)	56 (45.5)	215 (37.0)	
Non-Hispanic White	87 (19.0)	22 (17.9)	109 (18.8)	
Any other identity^c^	55 (13.1)	6 (4.9)	61 (10.7)	
US Census region				
Northeast	64 (14.0)	24 (19.5)	88 (15.2)	.21
Midwest	74 (16.2)	20 (16.3)	94 (16.2)	
South	209 (45.6)	59 (48.0)	268 (46.1)	
West	109 (23.8)	20 (16.3)	129 (22.2)	
Missing	2 (0.4)	0	2 (0.3)	
Urbanicity^d^				
Rural	108 (23.6)	37 (30.1)	145 (25.0)	.27
Suburban	191 (41.7)	49 (39.8)	240 (41.3)	
Urban	159 (34.7)	36 (29.3)	195 (33.6)	
Missing	0	1 (0.8)	1 (0.2)	
Education				
High school or less	105 (22.9)	40 (32.5)	145 (25.0)	.06
Some college or trade	153 (33.4)	31 (25.2)	184 (31.7)	
Bachelors or higher	200 (43.7)	52 (42.3)	252 (43.4)	
No. of comorbid conditions^e^				
0	234 (51.1)	52 (42.3)	286 (49.2)	.003
1	105 (22.9)	26 (21.1)	131 (22.5)	
2	63 (13.8)	16 (13.0)	79 (13.6)	
3	32 (7.0)	9 (7.3)	41 (7.1)	
4	9 (2.0)	9 (7.3)	18 (3.1)	
≥5	15 (3.3)	11 (8.9)	26 (4.5)	
Mean (SD)	1.1 (1.7)	1.5 (2.0)	1.2 (1.8)	.008
Health literacy needs^f^				
1 (Never)	309 (67.5)	44 (35.8)	353 (60.8)	<.001
2 (Rarely)	69 (15.1)	19 (15.4)	88 (15.1)	
3 (Sometimes)	50 (10.9)	34 (27.6)	84 (14.5)	
4 (Often)	14 (3.1)	17 (13.8)	31 (5.3)	
5 (Always)	14 (3.1)	9 (7.3)	23 (4.0)	
Missing	2 (0.4)	0	2 (0.3)	
Mean (SD)	1.6 (1.0)	2.4 (1.3)	1.8 (1.1)	<.001
Subjective numeracy (scale, 1-6), mean (SD)^g^	4.3 (1.3)	4.2 (1.2)	4.3 (1.3)	.35
Medical maximizing^h^				
1 (I lean toward waiting and seeing)	96 (21.0)	16 (13.0)	112 (19.3)	<.001
2	71 (15.5)	10 (8.1)	81 (13.9)	
3	102 (22.3)	16 (13.0)	118 (20.3)	
4	79 (17.2)	31 (25.2)	110 (18.9)	
5	55 (12.0)	25 (20.3)	80 (13.8)	
6 (I lean toward taking action)	55 (12.0)	25 (20.3)	80 (13.8)	
Mean (SD)	3.2 (1.6)	3.9 (1.6)	3.4 (1.7)	<.001
Disbelief in science (scale, 1-7), mean (SD)^i^	3.9 (1.4)	4.5 (1.4)	4.1 (1.4)	<.001
**Antibiotic outcome measures: for viral respiratory infection symptoms (ie, antibiotics not clinically indicated)**				
Likelihood of wanting antibiotics				
Mean (SD)	2.5 (1.0)	3.3 (0.9)	2.7 (1.0)	<.001
1 (Definitely would not)	90 (19.7)	9 (7.3)	99 (17.0)	<.001
2 (Probably would not)	125 (27.3)	9 (7.3)	134 (23.1)	
3 (Probably would)	151 (33.0)	47 (38.2)	198 (34.1)	
4 (Definitely would)	92 (20.1)	58 (47.2)	150 (25.8)	
Concern of adverse effects from antibiotics				
Mean (SD)	2.9 (1.4)	3.2 (1.4)	3.0 (1.4)	.06
1 (Not at all concerned)	95 (20.7)	19 (15.4)	114 (19.6)	.31
2 (Slightly concerned)	88 (19.2)	17 (13.8)	105 (18.1)	
3 (Somewhat concerned)	89 (19.4)	27 (22.0)	116 (20.0)	
4 (Moderately concerned)	93 (20.3)	27 (22.0)	120 (20.7)	
5 (Extremely concerned)	81 (17.7)	27 (22.0)	108 (18.6)	
6 (Not sure)^j^	12 (2.6)	6 (4.9)	18 (3.1)	
Self-reported antibiotic use in past 12 mo				
0 Times	458 (100)	NA	458 (78.8)	NA
3 Times	NA	69 (56.1)	69 (11.9)	
4 Times	NA	18 (14.6)	18 (3.1)	
5 Or more times	NA	36 (29.3)	36 (6.2)	

Abbreviation: NA, not applicable.

^a^ References for all survey measures reported in this study can be found in Supplement 1 along with full question wordings and response scales.

^b^ Groups were compared using the χ^2^ test (for proportions) and *t*-test (for means).

^c^ Includes respondents who reported their ethnicity as non-Hispanic and self-described their race as Jamaican (1 respondent), Middle Eastern (2 respondents), and multiracial (3 respondents).

^d^ Self-reported urbanicity grouped as: rural (“Rural” or “Small <100 000”), suburban (“Suburban near large city”), urban (“Mid-sized city (100 000 to 1 million),” “Large city >1 million”).

^e^ As captured by the Charlson Comorbidity Index (CCI). Maximum possible number is 12.

^f^ Ability to find, understand, and use information and services to inform health-related decisions as captured by the Single Item Literacy Screener (SILS). Higher scores correspond to needing more help reading health-related materials.

^g^ Individuals’ beliefs about their mathematical skills and their preferred presentation of numerical information as captured by the 3-item Subjective Numeracy Scale (SNS). Higher scores correspond to greater belief in their math skills.

^h^ Preferences for active vs. passive approaches to healthcare as captured by the single-item maximizer-minimizer elicitation question (MM1). Higher scores correspond to preferring active vs passive approaches to health care in clinically uncertain situations.

^i^ Tendency to negatively evaluate scientific methods, fields, authorities and the ideas they promote as captured by the Credibility of Science Scale (CoSS). Higher scores correspond to more negatively evaluating scientific methods, fields, authorities and the ideas they promote.

^j^ Respondents who gave an answer of not sure to the concern about side effects question were not included in the analyses for model 2.

Compared with nonusers, respondents with frequent use of antibiotics were younger (ages 50-64 years vs 18-33 years: aOR, 0.29; 95% CI, 0.16-0.53; *P* < .001), had more comorbid conditions (aOR, 1.14; 95% CI, 1.02-1.29; *P* = .02), needed more health literacy support (aOR, 1.54; 95% CI, 1.28-1.87; *P* < .001), preferred to act rather than watch and wait in uncertain medical situations (aOR, 1.22; 95% CI, 1.06-1.41; *P* = .005), and had greater disbelief in science (aOR, 1.26; 95% CI, 1.06-1.50; *P* = .009). Respondents with frequent antibiotic use were more likely than nonusers to want antibiotics for a viral respiratory infection (aOR, 1.64; 95% CI, 1.26-2.16; *P* < .001), but did not differ in their concern about adverse effects (Figure).

**Figure.**
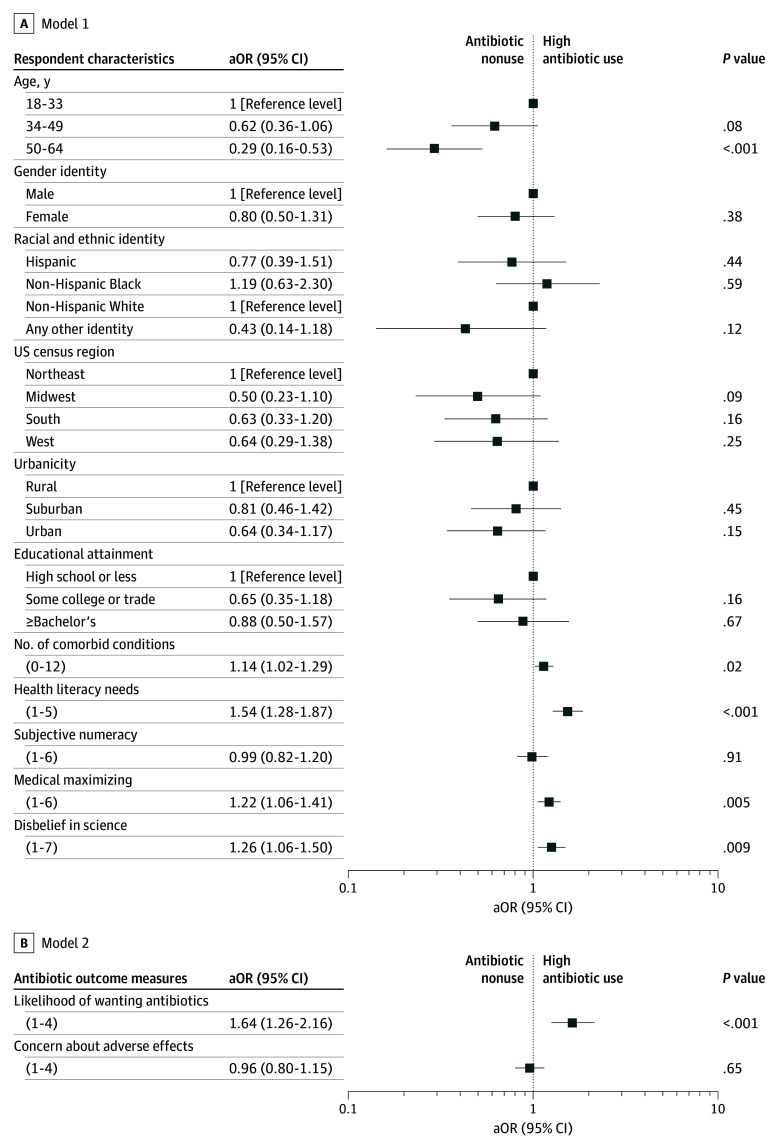
Exploratory Multiple Logistic Regression Analysis of Factors Associated With Antibiotic Utilization (Nonusers vs Frequent Use) Using a hierarchical approach model 2 (552 respondents; R2 Tjur = 0.21) also contained all variables included in model 1 (569 respondents; R2 Tjur = 0.18). Hosmer-Lemeshow Goodness-of-fit tests indicated good model fit for both models (model 1, χ^2^ = 13.08, *P* = .11; model 2, χ^2^ = 1.52, *P* = .99).

## Discussion

In this survey of US adults, compared with nonusers, adults with frequent antibiotic use reported needing more help reading health-related materials, preferred to act in uncertain medical situations, were more distrustful of science, and more likely to want antibiotics for a hypothetical viral respiratory infection. Limitations include potential inaccuracy of self-reported responses, lack of prescription context, and the underrepresentation of adults aged 65 years or more, non-English speaking individuals, and those without internet access.

These findings suggest important differences in cognitive beliefs and antibiotic preferences between nonusers and high antibiotic utilizing adults. Educational materials and interventions to promote appropriate antibiotic use should be tailored to the specific needs and concerns of patients who frequently use antibiotics.^4^

## Notes

Supplement 1. **eAppendix.** Survey Instrument and Methodological Details

Supplement 2.
Data Sharing Statement

## References

[1] Llor C, Bjerrum L. Antimicrobial resistance: risk associated with antibiotic overuse and initiatives to reduce the problem. Ther Adv Drug Saf. 2014;5(6):229-241. doi:10.1177/2042098614554919 25436105 PMC4232501

[2] Thorpe A, Sirota M, Juanchich M, Orbell S. Action bias in the public’s clinically inappropriate expectations for antibiotics. J Exp Psychol Appl. 2020;26(3):422-431. doi:10.1037/xap0000269 32271052

[3] Hicks LA, Bartoces MG, Roberts RM, . US outpatient antibiotic prescribing variation according to geography, patient population, and provider specialty in 2011. Clin Infect Dis. 2015;60(9):1308-1316. doi:10.1093/cid/civ076 25747410

[4] Spicer JO, Roberts RM, Hicks LA. Perceptions of the benefits and risks of antibiotics among adult patients and parents with high antibiotic utilization. Open Forum Infect Dis. 2020;7(12):ofaa544. doi:10.1093/ofid/ofaa544 33335939 PMC7731524

[5] Chua KP, Fischer MA, Linder JA. Appropriateness of outpatient antibiotic prescribing among privately insured US patients: *ICD-10-CM* based cross sectional study. BMJ. 2019;364:k5092. doi:10.1136/bmj.k5092 30651273 PMC6334180

[6] Scherer LD, Zikmund-Fisher BJ. Eliciting medical maximizing-minimizing preferences with a single question: development and validation of the MM1. Med Decis Making. 2020;40(4):545-550. doi:10.1177/0272989X20927700 32522094

