# Supplementary material for: Characteristics and Antibiotic Preferences of US Adults Reporting Frequent Use vs No Use of Antibiotics
Source: JAMA Netw Open. 2025 Mar 21;8(3):e251429. doi: 10.1001/jamanetworkopen.2025.1429 (PMC11929022; doi:10.1001/jamanetworkopen.2025.1429)
Supplement: Supplement 1. — eAppendix. Survey Instrument and Methodological Details [file jamanetwopen-e251429-s001.pdf]

Supplemental Online Content

Thorpe A, Lee RA, Fagerlin A, Vaughn VM, Szymczak JE. Characteristics and antibiotic preferences of US adults reporting frequent vs no use of antibiotics. *JAMA Netw Open.* 2025;8(3):e251429. doi:10.1001/jamanetworkopen.2025.1429

eAppendix. Survey Instrument and Methodological Details

This supplemental material has been provided by the authors to give readers additional information about their work.

© 2025 Thorpe A et al. *JAMA Network Open.*

eAppendix. Survey Instrument and Methodological Details

Supplement description

This supplement contains the specific survey quotas, references to survey items not included in the main text, additional methodological details, and the full list of survey items for the present study.

Survey Quotas

From March-April, 2024, respondents were recruited for this English-language survey by Dynata. Dynata are a commercial market research company with diverse pools of individuals who have agreed to be invited to take part in online survey studies (https://www.dynata.com/). This study was deemed exempt by the University of Utah IRB (IRB_00167676) with a waiver of informed consent granted and followed STROBE guidelines.

We used recruitment quotas for self-reported age, gender identity, racial/ethnic identity, and US census region to oversample underrepresented groups as follows:

**Overall sample recruitment quotas**	
Age in years	Gender identity
18-33 (22%)	Female (49%)
34-49 (22%)	Male (49%)
50-64 (22%)	Any other (1%)
≥65 (34%)	
Racial/Ethnic identity	US Census region*
Non-Hispanic White (30%)	Northeast (17%)
Non-Hispanic Black (30%)	Midwest (21%)
Hispanic (30%)	South (39%)
Any other (10%)	West (24%)

*US Census region quotas were chosen to reflect population estimates at the time of the survey according to https://www.census.gov/topics/population.html

References for survey measures

© 2025 Thorpe A et al. *JAMA Network Open*.

Number of comorbid conditions: Charlson Comorbidity Index (CCI).

Charlson ME, Pompei P, Ales KL, MacKenzie CR. A new method of classifying prognostic comorbidity in longitudinal studies: Development and validation. J Chronic Dis. 1987;40(5):373-383. doi:10.1016/0021-9681(87)90171-8

Health Literacy Needs: Single Item Literacy Screener (SILS).

- Morris NS, MacLean CD, Chew LD, Littenberg B. The Single Item Literacy Screener: Evaluation of a brief instrument to identify limited reading ability. BMC Fam Pract. 2006;7(1):21. doi:10.1186/1471-2296-7-21
- Chew LD, Bradley KA, Boyko EJ. Brief questions to identify patients with inadequate health literacy. Fam Med. 2004;36(8):588-594.

Subjective Numeracy: Subjective Numeracy Scale (SNS).

- Fagerlin A, Zikmund-Fisher BJ, Ubel PA, Jankovic A, Derry HA, Smith DM. Measuring numeracy without a math test: development of the Subjective Numeracy Scale. Med Decis Making. 2007;27(5):672-680. doi:10.1177/0272989X07304449
- McNaughton CD, Cavanaugh KL, Kripalani S, Rothman RL, Wallston KA. Validation of a Short, 3-Item Version of the Subjective Numeracy Scale. Med Decis Mak Int J Soc Med Decis Mak. 2015;35(8):932-936. doi:10.1177/0272989X15581800

Medical Maximizing: single-item maximizer-minimizer elicitation question (MM1).

Scherer LD, Zikmund-Fisher BJ. Eliciting medical maximizing-minimizing preferences with a single question: development and validation of the MM1. Med Decis Making. 2020;40(4):545-550. doi:10.1177/0272989X20927700

Disbelief in science: Credibility of Science Scale (CoSS).

Hartman RO, Dieckmann NF, Sprenger AM, Stastny BJ, DeMarree KG. Modeling attitudes toward science: development and validation of the credibility of science scale. Basic Appl Soc Psychol. 2017;39(6):358-371. doi:10.1080/01973533.2017.1372284

Additional methodological details

© 2025 Thorpe A et al. *JAMA Network Open*.

Completion rate.
The overall completion rate reported reflects the total number of respondents who completed the survey out of all the eligible respondents who started it (1560 out of 1738, 89%).

Missingness
We did not perform any imputation methods because the missingness across study variables was infrequent and very low when present (<0.3% at most). Rates of missingness across study variables are reported in Table 1.

Race and Ethnicity.
The race and ethnicity options pre-programmed into the survey were defined by the investigators but allowed all respondents the opportunity to self-describe if they wished. Race and ethnicity were included to allow us to describe the sample and examine variation in perceptions by demographic characteristics.

Survey items

Study information:
We invite you to participate in a research study about decisions you make regarding your health. In this study, you will be asked some questions about your personal opinions, previous decisions, and experiences regarding antibiotics, common infections, and COVID-19. We will also ask some questions about yourself. If you agree to participate, we would like you to answer the questions on the following screens.

It will take approximately 15 to 20 minutes to complete this survey. You are free to skip any questions that you prefer not to answer. Compensation for participation will be provided in accordance with your panel agreement.

Every effort will be made to protect your privacy and confidentiality. We will not collect your name or any identifying information about you. Your participation will be completely anonymous and it will not be possible to link you to your responses.

This study is not designed to benefit you directly. You have a choice about being in this study. You do not have to be in this study if you do not want to be.

The data we collect will be used for this study but may also be important for future research. Your data may be used for future research or distributed to other researchers for future study without additional consent if information that identifies you is removed from the data.

© 2025 Thorpe A et al. *JAMA Network Open*.

Taking part in this research study is completely voluntary. If you do not wish to participate in this study, simply click in the corner to close the web browser window.

You may have questions about your rights as someone in this study. If you have questions, you can call the University of Utah Institutional Review Board (the responsible Institutional Review Board) at 801-581-3655. Questions or concerns about this study or interest in the final results may be directed to Dr. Valerie Vaughn at valerie.vaughn@hsc.utah.edu. Thank you for taking part in this study.

Demographics:
What is your age?

Skip logic: if answer is <18 then skip to End of Survey.

How would you describe your gender identity?
- Female (1)
- Male (2)
- Transgender woman/Transwoman (3)
- Transgender man/Transman (4)
- Non-Binary/Third gender (5)
- Prefer not to say (6)
- Other please specify (7)

How would you describe your ethnic group or background?
(Please select all that apply)
- American Indian or Alaskan Native (1)
- Asian or Asian American (2)
- Black or African American (3)
- Native Hawaiian or other Pacific Islander (4)
- White or European American (5)
- Other please specify (6)

Are you Hispanic or Latino/a or Latinx?
- No (1)
- Yes (2)

What state do you live in?

© 2025 Thorpe A et al. *JAMA Network Open*.

↓ Alabama (1)…Wyoming (52)

Please read the text below carefully and imagine that the situation described is real.

For over a week you have been feeling really ill and experiencing flu-like symptoms. You have had a couple of headaches and at one time had a high temperature (around 102ºF). Repeated dry chesty coughing has caused you to develop a sore throat. Throughout the day your nose goes back and forth between being runny or blocked and you have been sneezing a lot. The symptoms persist during the day and throughout the evening. Your appetite is low, but you are still able to drink water and take small bites of food. Since the symptoms started, they have not really improved. You have taken a test which shows that **you do not** have COVID-19.

Would you want to get antibiotics for these symptoms?
- Definitely WOULD NOT want to get antibiotics (1)
- Probably WOULD NOT want to get antibiotics (2)
- Probably WOULD want to get antibiotics (3)
- Definitely WOULD want to get antibiotics (4)

How concerned are you about experiencing short-term side effects from taking antibiotics?
- Not at all concerned (1)
- Slightly concerned (2)
- Somewhat concerned (3)
- Moderately concerned (4)
- Extremely concerned (5)
- Not sure (6)

Now we would like to learn a little more about you. Thinking about your own personal history, experiences, and beliefs, please indicate your response to the following statements:

Sometimes, medical action is clearly necessary, and sometimes it is clearly NOT necessary. Other times, people differ in their beliefs about whether medical action is needed. In medical situations where it's not clear, do you tend to lean towards taking action or do you lean towards waiting and seeing if action is needed? Importantly, there is no "right" way to be. Please answer on the 1-6 scale below:

© 2025 Thorpe A et al. *JAMA Network Open*.

- I lean toward waiting and seeing (1)
- (2)
- (3)
- (4)
- (5)
- I lean toward taking action (6)

© 2025 Thorpe A et al. *JAMA Network Open*.

Please indicate how much you agree or disagree with each statement. There are no right or wrong answers. Please answer in a way that reflects your own personal beliefs:

	Strongly disagree (1)	Disagree (2)	Somewhat disagree (3)	Neither agree nor disagree (4)	Somewhat agree (5)	Agree (6)	Strongly agree (7)
People trust scientists a lot more than they should (1)	○	○	○	○	○	○	○
People don't realize just how flawed a lot of scientific research really is (2)	○	○	○	○	○	○	○
A lot of scientific theories are dead wrong (3)	○	○	○	○	○	○	○
Sometimes I think we put too much faith in science (4)	○	○	○	○	○	○	○
Our society places too much emphasis on science (5)	○	○	○	○	○	○	○
I am concerned by the amount of influence that scientists have in society (6)	○	○	○	○	○	○	○

© 2025 Thorpe A et al. *JAMA Network Open*.

Thinking about your own personal medical history, please indicate your response to the following statements:

In the past 12 months, I have taken antibiotics...
- 0 times (1)
- 1 time (2)
- 2 times (3)
- 3 times (4)
- 4 times (5)
- 5 or more times (6)

As far as you know, do you have any of the following health conditions at the present time?

	No, I do not have this condition (0)	Yes, I have this condition (1)
Asthma, emphysema, or chronic bronchitis, COPD *(other lung disease)* (1)	○	○
Arthritis or rheumatism (2)	○	○
Cancer, diagnosed in the past 3 years (3)	○	○
Diabetes (4)	○	○
Digestive problems *(such as ulcer, colitis, or gallbladder disease)* (5)	○	○
Heart trouble *(such as angina, congestive heart failure, or coronary artery disease, having a past heart attack)* (6)	○	○
HIV illness or AIDS (7)	○	○
Kidney disease (8)	○	○
Liver problems (such as cirrhosis) (9)	○	○
Stroke (10)	○	○
High blood pressure (hypertension) (11)	○	○
Very overweight or obese (12)	○	○

© 2025 Thorpe A et al. *JAMA Network Open*.

What is the highest level of schooling you have completed? (Check one)

- None (1)
- Elementary school (2)
- Some high school but no diploma (3)
- High school (Diploma or GED) (4)
- Some college, but no degree (5)
- Trade school (6)
- Bachelor’s degree (BS, BA, etc.) (7)
- Master’s degree (MA, MPH, etc.) (8)
- Doctoral/Professional degree (PhD, MD, etc.) (9)

How would you best describe the place where you live?

- Rural (1)
- Small city, e.g. less than 100,000 people (2)
- Suburban, near a large city (3)
- Mid-sized city, 100,000 to 1 million people (4)
- Large city, more than 1 million (5)
- Other, please specify (6)

Please take a moment to answer some questions about how you feel about reading health information. Please give the response that best matches how you feel.

How often do you have someone (like a family member, friend, hospital/clinic worker or caregiver) help you read instructions, pamphlets or other written health materials from your doctor or pharmacy?

- Never (1)
- Rarely (2)
- Sometimes (3)
- Often (4)
- Always (5)

© 2025 Thorpe A et al. *JAMA Network Open*.

How good are you at working with fractions?

- Not good at all (1)
- (2)
- (3)
- (4)
- (5)
- Extremely good (6)

How good are you at figuring out how much a shirt will cost if it is 25% off?

- Not good at all (1)
- (2)
- (3)
- (4)
- (5)
- Extremely good (6)

How often do you find numerical information to be useful?

- Never (1)
- (2)
- (3)
- (4)
- (5)
- Very often (6)

Debrief: Thank you for your participation! You are now finished with this survey. In this study, we are interested in understanding how to communicate about the risks of antibiotic resistance and people's attitudes and understanding of antibiotics. We greatly appreciate all your responses! We are learning a lot about these topics, and we hope to share our findings broadly soon.

For accurate, up-to-date information please see the following CDC websites:

- Antibiotic resistance: https://www.cdc.gov/drugresistance/index.html
- Patient resources about antibiotics: https://www.cdc.gov/antibiotic-use/materials-references/index.html
- COVID-19: https://www.cdc.gov/coronavirus/2019-ncov/index.html

Thank you for participating in this survey.

© 2025 Thorpe A et al. *JAMA Network Open*.
