# Supplementary material for: Characteristics and Antibiotic Preferences of US Adults Reporting Frequent Use vs No Use of Antibiotics
Source: JAMA Netw Open. 2025 Mar 21;8(3):e251429. doi: 10.1001/jamanetworkopen.2025.1429 (PMC11929022; doi:10.1001/jamanetworkopen.2025.1429)
Supplement: Supplement 2. — Data Sharing Statement [file jamanetwopen-e251429-s002.pdf]

Data Sharing Statement

Thorpe. Characteristics and Antibiotic Preferences of US Adults Reporting Frequent Use vs No Use of Antibiotics. *JAMA Netw Open*. Published March 21, 2025.
doi:10.1001/jamanetworkopen.2025.1429

Data
Data available: Yes
Data types: Deidentified participant data, Data dictionary
How to access data: Thorpe A, Lee RA, Szymczak JE, Fagerlin A, Vaughn VM. Psychological Research Insights for Messaging Effectively About Antibiotics (PRIME). https://osf.io/h26jp/?view_only=8b3362ee3cb64f08965ef25474d3af6a. Published November 1, 2024.
When available: With publication

Supporting Documents
Document types: None

Additional Information
Who can access the data: Anyone requesting the data
Types of analyses: For any purpose
Mechanisms of data availability: Without investigator support
